# Children’s understanding of epilepsy: A qualitative study

**DOI:** 10.1016/j.yebeh.2021.107994

**Published:** 2021-07

**Authors:** Jeni Harden, Rebecca Black, Martyn Pickersgill, Jay Shetty, Ailsa McLellan, Celia Brand, Michelle Small, Jane McDonnell, Lorna Clarke, Richard F. Chin

**Affiliations:** a Usher Institute, The University of Edinburgh, UK; b Muir Maxwell Epilepsy Centre, Centre for Clinical Brain Sciences, The University of Edinburgh, UK; c Department of Paediatric Neurosciences, Royal Hospital for Sick Children, Edinburgh, UK; d Borders General Hospital, Melrose, UK; e Child Life and Health, MRC Centre for Reproductive Health, The University of Edinburgh, Edinburgh, UK

**Keywords:** Children, Epilepsy, Understanding, Qualitative, Animation, Information

## Abstract

**Purpose:**

To use a qualitative research approach to determine children’s understandings of epilepsy and their epilepsy treatment.

**Methods:**

Children aged 7–16 years with physician-confirmed active epilepsy (i.e., having had an epileptic seizure in the past year and or currently taking antiepileptic drugs (AEDs), and not known to have an intellectual disability, were invited to participate. Children had semi-structured interviews separately on two occasions. Between the first and second interviews, an observation of a routine epilepsy clinic appointment of individual children was conducted, and was then discussed during the second interview. Participatory research tools were used in both child interviews to facilitate discussions. Interviews were audio recorded and transcribed, pseudonymized and entered into NVivo (version 12, QSR International). Data were analyzed using a thematic approach.

**Results:**

Twenty-three children of mean age 10.1 years (range 8–14), mean duration of epilepsy of 4.6 years (range 2–10) were enrolled. Twelve were 12 female; 7 had focal, 14 had generalized, and 2 had combined epilepsy; 20 were on monotherapy; and 16 had tried previous AEDs. All had an initial (first) interview; 20 were observed during a clinic appointment and had a second interview. Five broad themes emerged: understanding of epilepsy; understanding of seizures; understanding of medication; understanding of children’s role in clinical appointments; influences on children’s understanding. Children spoke about what epilepsy meant by describing the physical sensations of having a seizure or through the act of taking medication. Children described the role they had, or felt they should have, but reported challenges in being meaningfully involved in clinical appointments. While healthcare professionals were initial information nodes, epilepsy information from parents appeared to be more significant for children.

**Conclusions:**

The perspectives of children with epilepsy are valuable for clinicians to understand; assumptions should not be made that children’s views can be accessed via parents. Clinicians need to be constantly aware of children’s views and ways of understanding and communicating about their epilepsy. To support this, the research – drawing on children’s words, meanings, and stories – was used to inform an easily accessible, gender-neutral, animation about epilepsy that provides information about the condition, seizures, and medication (https://youtu.be/MO7xXL2ZXP8).

## Highlights

• Children’s understanding of epilepsy reflects their lived experiences.
• Parents are key information gatekeepers and shape children’s understanding.
• Children face challenges in being meaningfully involved in healthcare decisions.

## 1 Introduction

An estimated 0.5–1% of children and young people worldwide have epilepsy [1]. Epilepsy can have short- and long-term effects and implications for both children and their families, with a range of professionals involved in diagnosis, treatment, and care [2]. Consequently, a diagnosis of epilepsy holds a variety of implications for children with the condition, as well as their families, beyond the physical effects associated with seizures [3], [4]. As such, it is essential to engage children with epilepsy about their care and the management of the condition.

To-date, much attention has been focused on seeking the perspectives of parents about what they perceive are their child’s thoughts and feelings about epilepsy; the Quality of Life Childhood Epilepsy (QOLCE) questionnaire, for example, is completed by parents. Yet, children’s experiences of epilepsy can differ from the assumptions of their parents, and researchers and clinicians can, and should pay heed to their views and accounts [5], [6]. While some qualitative research has sought to gain children and young people’s own views and experiences of their epilepsy [6], [7], [8], [9], [10], [11], [12], systematic reviews have highlighted that significant gaps remain in the literature [10], [13]. In particular, despite recognition of the importance of listening to, and consulting with children regarding their healthcare [14], younger children’s accounts of their epilepsy and involvement in associated care are under-examined [15].

This research gap has implications for clinical practice. Obtaining insights into children’s understanding of their condition, and their experiences of negotiating care and their role in their own care, can offer insights into the practical implications for children of managing their epilepsy. Accessing children’s experiences directly can lead to a deeper understanding of how epilepsy shapes children’s lives and can contribute to the development of improved resources and interventions [15]. Furthermore, increasing children’s involvement in their care and clinical decision-making can enhance and promote their agency and autonomy, with the additional benefit that they are better equipped to deal with the emotional and practical complexities of living with a chronic illness [14]. This is crucial for both the success of medical treatments and interventions, and the well-being of children with epilepsy.

In this paper we report findings from a qualitative study exploring children’s experiences of living with childhood epilepsy. Specifically, we consider (1) What are children’s understandings of epilepsy, seizures, medication, and their role in clinical appointments? (2) What are the implications of the findings for clinical practice?

## 2 Methods

### 2.1 Recruitment

Study inclusion criteria were: (1) children aged 7–16 years with physician-confirmed active epilepsy (i.e., having had an epileptic seizure in the past year and or currently taking antiepileptic drugs (AEDs) [16], (2) English as first or primary spoken and written language; (3) diagnosed with epilepsy at least two years prior to recruitment; and (4) not known to have an intellectual disability on previous formal cognitive assessment, nor any physician concerns about possible intellectual disability irrespective of whether the child was awaiting formal cognitive assessment or not. We defined socioeconomic status of individuals as high if their home postal code corresponded to Scottish Index of Multiple Deprivation Quintile 1–3, and low if it was 4–5 [17]. The age range of 7–16 years was selected as fewer studies regarding chronic illness have included children between those ages, with even fewer obtaining children’s own self-reports and accounts [18]. Originally, the age range of those to be included was 7–11 years, with the aim of focusing on ‘middle childhood’, however, slow initial recruitment sparked a need to increase the age range to include those up to age of 16 years to boost recruitment.

Children who fulfilled the inclusion criteria were invited for study entry from attendance to pediatric epilepsy clinics at two hospital sites: one which provides secondary and tertiary pediatric epilepsy services, and the other providing secondary services (we do not include details of the hospital sites in order to enhance participant anonymity). To ensure confidentiality and adherence to data protection regulation, an opt-in recruitment was adopted. First contact with potential participants was made by healthcare professionals (consultant pediatricians/nurse specialists/nurse consultants) with expertise in epilepsy, who were part of a potential participant’s established medical team. The health professionals provided a recruitment pack that included: a study invitation letter, a child information sheet, a parent information sheet, and an opt-in form.

Upon receipt of the opt-in form by the research team (via post, email, telephone call, or handed-in), the family was contacted after a ‘cooling off’ period of at least 48 h to arrange a meeting with the child and parents to discuss the study in greater detail. At that meeting, potential involvement in the study was discussed with the child and their parents. This included discussion of the research tools that would be used, what would happen in the observation, and confidentiality during the research process.

At the end of this initial meeting, preliminary written (age appropriate) consent was given by children and their parents. Whenever an interview or observation was undertaken, consent was verbally reviewed on each occasion to confirm that it remained in place and that all participants – i.e., both children and their parents – were happy to continue in the study. In other words, consent was considered to be on-going process.

### 2.2 Data collection

There were three points of data collection with children – (1) a semi-structured interview; (2) Observation of a routine clinic appointment; (3) a semi-structured interview. The second interview was carried out to coincide with the consecutive regular outpatient epilepsy clinic appointments, so interviews were conducted on average four to six months apart. The interviews and observation was conducted by RB who was not part of the clinical team. Parents were also interviewed separately on two occasions, but here we focus on data from the children.

#### 2.2.1 Interviews with children

Interview topic guides were developed following informal discussions with children with epilepsy, parents, and healthcare professionals. The topic guides were intended to help direct the flow of the interview discussions, providing initial questions and follow up probes (See supplementary information S1). The first interview explored how children made sense of, and experienced epilepsy in their everyday lives. The focus of the children’s second interview was the observed clinic appointment, and their own and others’ involvement in it; children were asked to reflect on the time before the appointment, the time during, and what would normally happen afterward the appointment.

The first interviews took place either at the family home or in a quiet room at the University of Edinburgh, whichever was more convenient to the family. Subject to agreement between the child, their parents, and RB, children were interviewed alone with their parents in a nearby room. The majority of children asked their parent to leave the room, though the door was always left open. The second interview usually took place in the hospital directly after the routine epilepsy clinic consultation that was observed. When this was unfeasible, the interviews were again conducted in the family home. In the clinic, a quiet office was used for the interview and both children and parents were made to feel as comfortable as possible in the space (including through the provision of drinks and snacks). As with the first interview, children and parents were asked if they wished the other to be in the room with them during their interview. All but three children elected to have their parent sit with them for this second interview.

Children were provided with paper ‘up and down thumbs’ to use if they felt uncomfortable or did not wish to answer a question. If the thumb was turned down, the interviewer would change the subject; if the thumb was used again, the participant was asked whether they wished to continue or take a break. This tool was used to facilitate on-going consent; some children made use of this to draw a particular topic of discussion to a close, though none chose to stop the interview.

In the interviews with children, additional research tools were also used in order to facilitate children’s engagement with the interview and provide them with alternative modes of communication beyond their verbal contributions. A brief outline is provided below and further information is provided as a supplementary document (S1).

• *Spider diagrams:* children were given an A3 sheet of paper with the word ‘epilepsy’ written in a circle in the middle, and each child was asked to draw legs on to this central ‘spider’ with all their thoughts, experiences, and feelings about epilepsy.
• *Magnetic families and friend’s tool* [19]: children were given blank magnets, and asked to think of the people in their family who help look after them, and either draw a picture of them or write each person’s name on each magnet, including one for themselves. The magnets were then placed on a board, where they could be moved around.
• *Comic book vignettes*: Vignettes provide concrete examples about individuals, situations, and structures that participants can offer their thoughts on [20]. Three comic strip vignettes were used to explore the children’s experiences of epilepsy and associated treatment and management regimen.
• *‘Pots and beads’ ranking exercise:* This was used in the second interview to explore children’s feelings about and involvement in the clinic appointment. The children chose different colored pots to represent each of these individuals in the clinic appointment and distributed the beads among the various pots, from most to least, in response to statements such as: speaking in appointments, asking questions, and making decisions.

#### 2.2.2 Observation of routine epilepsy clinic appointment

The clinic appointments formed a standard part of pediatric epilepsy care in South East Scotland. They serve as ‘check-ups’ to ensure treatment regimen are working effectively and provide the opportunity for children and parents to discuss any issues or concerns they have regarding the protocols, and epilepsy more generally. The observations of the clinic appointment were unobtrusive, with RB quietly sitting away from the participants and healthcare professionals making discreet notes on topics being discussed, and with regard to the interaction between, and the engagement and involvement of the various attendees. The researcher took notes to include: ‘content’ (what is being discussed), ‘engagement’ (levels of engagement), ‘interactions’ (how and between who), ‘questioning’ (how and between who), ‘presentation’ (how are questions/information being discussed). Observation notes were typed up after the observed clinic appointments and handled electronically with similar techniques for anonymity and confidentiality as described for the interview. By combining observation in the clinic with a further interviews, it was possible to discuss the observations and gain additional insight into the child’s experience of the appointment.

### 2.3 Data analysis

The interviews were audio recorded and transcribed fully. All identifying features were deleted from transcripts, and each transcript was allocated a unique participant code identifier. The link between the patient identifiable information and unique code identifiers were stored in a password protected file. Furthermore, to protect patient confidentiality, RC – a member of the research team who was also a pediatrician with expertise in epilepsy – had no access to anonymized transcripts.

Thematic analysis of the qualitative data (interviews and observational) was undertaken by RB, in ongoing dialogue with JH and MP, using an approach informed by the Framework analytic method [21], [22]. This approach supported a process of familiarization of the data, summarization, description, and explanation; this was useful in ensuring that we retained a close tie to participants’ contributions, while facilitating interpretation of the data. All interviews were transcribed verbatim, pseudonymized, and entered into NVivo qualitative data analysis software (version 12, QSR International) to facilitate data management and coding, and the generation of themes. Transcripts were subject to repeated reading and comparison to identify recurrent issues, including those not foreseen at the study’s outset. A coding framework, which captured both the original research questions and emergent issues, was developed and applied to the qualitative interview data. Thematic charts were then developed from the coded data; each chart detailed a code or sub-code, with descriptions of each individual participant’s responses to it in the rows. Reading in and across the charts, the data were subject to further analysis to identify and explore linked ideas and patterns, leading the construction of themes and sub-themes.

### 2.4 Ethics

The study was given a favorable ethical opinion (14/SS/0090) by the South East Scotland NHS Research Ethics Committee.

## 3 Results

### 3.1 Participant demographics

Twenty-nine children and parents initially expressed an interest in participating. Following a cooling-off period of two working days after the initial meeting, four families chose not to take part and two were uncontactable.

Twenty-three children of mean age 10.1 years [8], [9], [10], [11], [12], [13], [14], mean duration of epilepsy of 4.6 years [2], [3], [4], [5], [6], [7], [8], [9], [10] were enrolled. Twelve were 12 female; 7 had focal, 14 had generalized and 2 had combined epilepsy; 12 had high socioeconomic status; 20 were on monotherapy; and 16 had tried previous AEDs (Table 1). All had an initial (first) interview. Three children did not continue to the observation and second interview stage due to changes in their circumstances.

**Table 1:** Participant characteristics.

Study Number	Age	Gender (M/F)	Age at Diagnosis	Epilepsy Type	Seizure Types	Current AEDs (*N*)	Previous AEDs (*N*)
P1	10	M	2	Genetic generalized	Tonic-Clonic and myoclonic	1	1
P2	10	F	5	Structural focal	Tonic, Tonic-clonic	1	1
P3	9	M	5	Genetic combined generalized and focal	Tonic-clonic, tonic	1	1
P4	11	F	6	Childhood absence epilepsy	Absence	1	3
P5	10	M	7	Childhood epilepsy with centrotemporal spikes	Hemifacial clonic	1	0
P6	10	M	1	Frontal lobe	Somatosensory, asymmetric tonic, myoclonic	1	0
P7	8	F	1	Genetic generalized	Tonic-Clonic	1	2
P8	13	M	9	Genetic generalized	Tonic-Clonic	1	0
P9	13	F	3	Temporal lobe epilepsy	Automatisms, Tonic-Clonic	3	2
P10	9	F	6	Childhood absence epilepsy	Absence, tonic-clonic, myoclonic	2	4
P11	11	F	8	Juvenile absence epilepsy	Absence, Tonic-Clonic	2	1
P12	9	F	6	Focal epilepsy of unknown cause	Focal to bilateral tonic-clonic	1	0
P13	8	F	6	Temporal lobe epilepsy	Sensory aware	1	1
P14	11	F	6	Focal epilepsy of unknown cause	Focal to bilateral tonic-clonic and absence	1	2
P15	14	F	4	Focal epilepsy of unknown cause	Focal to bilateral tonic-clonic and absence	1	2
P16	10	M	7	Genetic generalized	Myoclonic	1	2
P17	8	M	6	Focal epilepsy of unknown cause	Focal to bilateral tonic-clonic and absence	1	1
P18	8	M	6	Childhood absence epilepsy	Absence	1	1
P19	10	F	7	Childhood epilepsy with centrotemporal spikes	Hemifacial clonic	1	0
P20	11	M	7	Childhood absence epilepsy	Absence	1	1
P21	11	M	8	Childhood absence epilepsy	Absence	1	0
P22	11	F	6	Childhood epilepsy with centrotemporal spikes	Hemifacial clonic	1	0
P23	8	F	5	Childhood absence epilepsy	Absence	1	1

### 3.2 Themes

Thematic analysis identified five broad themes, presented below: understanding of epilepsy; understanding of seizures; understandings of medication; understanding of children’s role in clinical appointments; influences on children’s understanding (Table 2). Quotes from the interviews are presented using their participant number.

**Table 2:** Themes and Sub-themes.

Theme	Sub-Theme
Understanding of epilepsy	Epilepsy as brain malfunctioning
	Epilepsy as treatment
	Epilepsy as seizure occurrence
	Difficulty describing epilepsy

Understanding of seizures	Unpredictability
	Embodied experiences
	Absence of awareness
	Other people’s descriptions
	Emotional reactions to seizures
	Reluctance to speak about seizures

Understanding of medication	Form of medication
	Purpose of medication
	Size, texture, and taste of medication
	Side effects

Understanding of children’s in clinical appointments	Sharing responsibility with parents
	Not understanding terminology and meanings
	Reluctance to discuss seizures
	Feeling silenced by adults

Influences on children’s understanding	Information from healthcare professionals
	Parental influence
	Other children with epilepsy

### 3.3 Understanding of epilepsy

#### 3.3.1 Epilepsy as brain malfunctioning

Some of the children captured the involvement of the brain in their understanding of epilepsy, in general terms:

“about the brain, I think” (P2)

“it’s like problems that goes with your brain and stuff” (P11)

“it’s a sickness. A sickness of the brain” (P9)

Other children incorporated more detailed descriptions of the malfunctions of the brain in their accounts of what epilepsy was. For example:

“I think you have a little bit of… little, little bit of electric things going through your brain and then sometimes they get hyper I think, like, and then that's what makes you have a fit” (P21)

“Basically, there is something in the brain stopping, erm some stopping erm some objectives getting to the right part of the brain so it erm it makes you act in different ways because it’s not got part of its constructions” (P8)

#### 3.3.2 Epilepsy as treatment

It was rare for children to use explicitly physiological language in their reflections; instead, participants tended to connect their understanding of epilepsy to their treatment regimen. For example, one child said,

“It means you're on medicine and it means, like, if you don't take your medicine you'll get, like, a sore head if you don't take your tablets” (P7).

She went on to draw a picture of her tablets, describing the drawing as:

“It's me and there's a tablet… and there's my little tablets. That’s what it is. Epilepsy”.

Responses such as these indicate how the children conceptualized and understood epilepsy through a tangible aspect of the condition, namely taking medication.

#### 3.3.3 Epilepsy as seizure occurrence

Some children equated epilepsy with seizures: “it’s fits”(P12); “it’s like having episodes” (P20). Others also explained epilepsy through descriptions of their seizures. For example

“well I would say that epilepsy is like… er, violently vibrating [shakes arms in demonstration]” (P22)

“it’s a party you have in your head” (P17)

One boy repeated the metaphor his Mum had told him, to explain epilepsy as seizures:

“my mum tells me it fizzes. My head” (P1).

#### 3.3.4 Difficulty describing epilepsy

Several of the children struggled to explain or describe what they understood by, or knew about epilepsy. The following quote exemplifies the challenges associated with narrating such a complex condition:

“Well it means like… it just means something that I know. Like, that might happen. So, like I know something might happen to me or something. So, like… I can… can’t really describe it. […] I don’t know the words to say it”. (P3)

These challenges also related to the ephemerality and, for some, the subjective immateriality of epilepsy:

“I don’t really feel the effect of it. You know?” (P18).

Even when drawing on the explanations of others, the children sometimes reported difficulty in understanding. The boy (P1) above who reported his mother’s explanation of epilepsy as fizzing in his head, went on to say:

“I don’t really get what it means, fizz? I’m thinking fizz as like when you shake a fizzy, like, a lemonade and then you open the thing and that sort of fizzy, but I don’t know what sort of fizzy means?” (P1)

Despite the difficulty of articulating their understanding of epilepsy, most of the children appeared quite content with the nature of their understandings of epilepsy. This was even the case when some participants reported what they described as an absence of understanding, as one said “I don’t really understand it…. I don’t really want to.” (P5)

Nevertheless, there were some children who described a desire for more information on epilepsy:

“I think I would like to know it more. Like properly”. (P20)

“I understand mostly. But more would be good, helpful”. (P14)

### 3.4 Understanding of seizures

#### 3.4.1 Unpredictability

Many of the children described seizures as unexpected: “It just happens” (P2) and “it surprises me” (P6). Seizures just happen, they often cannot be planned for, though some may be aware of triggers. Even when some children became aware of specific triggers, as discussed above, there can be uncertainty about when they happen. For example, despite knowing that his ‘shakes’ are triggered by loud noises and becoming startled, one still spoke about the unexpected nature of having one:

“Well sometimes they just happen at school, just randomly, but when like the teacher shouts at someone or, say, she shouted at me or something, I'd get a wee… I might get a wee bit of a fright and then start, like, having a wee shake” (P16)

#### 3.4.2 Embodied experiences

Many children were able to describe what they considered happened during a seizure. Children’s discussions centered on physical descriptions of what they perceived happens to their body as they experience a seizure.

“I go all dizzy and then I normally go to sleep and then when I wake up I can’t talk, and I feel sick and I get all numb”. (P15)

The head and eyes were frequently specifically mentioned by children as key aspects of their descriptions of seizure experiences.

“Er… Your eyes go funny. […] They go like that way [he diverts his eyes left and stares into the distance]”. (P20)

“It's like a feeling in… it's almost like a head rush that doesn't tickle”. (P22)

#### 3.4.3 Absence of awareness

In contrast to those who described the embodied experience of seizures, several children discussed a lack of something happening during a seizure.

“when you’re having it, you feel… you just, feel, nothing”. (P3)

“It’s when you stay up for a bit and then you just wipe out and want to fall asleep and so. And then you when wake up you don’t know what happened, so you fall asleep”. (P11)

#### 3.4.4 Other people’s descriptions

Perhaps because of this lack of awareness of what happens during the seizure, some children spoke about their experiences through descriptions others had provided for them. For example:

“Mummy and daddy say I make a weird sound”(P9).

“Apparently I either, like, fall on the floor or do something like that” (P22).

Some children had questions regarding their seizures and what happens during them

“Like, how hot… how hot do I get? Like, I don’t know if I, like, start to sweat and stuff” (P3)

“What I look like when I have one, what do I do when I have one and what do I look like when I have one, am I looking sad or am I looking happy or am I looking straight faced? It’s because I can't see myself” (P10)

#### 3.4.5 Emotional reactions to seizures

Many of the children described their feelings toward seizures. Most commonly, they described a sense of being scared about the potential of having a seizure, or after experiencing a seizure:

“I'll just be like a bit scared and… don't know” (P2)

Here one child described her feelings of anger

“I’m maybe even angry with them…Because they feel horrible and they're not nice and it's like a horrible child at school, it's like if… a horrible child at school makes you feel horrible and isn't nice they make me angry” (P22).

Several others also described them as embarrassing. For example,

“well it’s just that you never know when it’s going to happen so…” going on a moment later to explain: “well if you could like have… like a watch to say you going to have one you know or something… then I could not go to school so I wouldn’t get embarrassed and stuff” (P4)

#### 3.4.6 Reluctance to speak about seizures

However, not all children spoke about their experiences of seizures. For example, two girls both seemed unsure how to describe the experience and simply stated, “I don’t know” (P13 and P19) to questions about what their seizures felt like; neither wished to draw a picture. When asked about their seizures, some also said, or indicated, that they did want to discuss it, highlights the discomfort some children might experience in talking about seizures.

“[epilepsy] is quite scary… I don’t like talking about it” (P15).

“I don’t want to er… [turns thumb down] better forgetting them”. (P9)

### 3.5 Understanding of epilepsy medications

We noted above that medication formed a central part of children’s understanding of epilepsy. In this section, we turn to a consideration of how the children discussed their treatment regimen.

#### 3.5.1 Form of medication

Most of the children spoke of their medication in terms of its preparation (e.g., liquid, tablet) or, less frequently, by its color. For example, one girl described the different medicines she has taken:

“the liquid one was first. Then there were the two of the tablets ones… one was a capsule tablet thing” (P4).

All children were able to give some form of measurement as an indicator of their daily medication dosage. Some gave a detailed description of their dosage, for example: “it’s 10mls a day, so 5 in the morning and 5 in the evening”(P8), while others gave less precise measurements; e.g., “I take two spoons” (P6).

#### 3.5.2 Purpose of medication

Most children described why they were taking medication, the relative importance of doing so, and what the desired effects were. The majority spoke about their medication in terms of its effect, for instance: “it stops my headaches” (P7) and “they are stopping me having seizures” (P14). These children explained their medication through its impact on their seizures, or rather their lack of seizures.

This idea of medication ‘stopping’ seizures was also evident in the children’s response to Ben’s story (one of the three comic book vignettes used during the first interview). Many children linked the importance of medication to the consequences of Ben refusing to take them. For example,

“I'll say, 'Ben you need to take your medicine or you'll get a sore head'” (P7)

#### 3.5.3 Size, texture, and taste of medication

In discussing taking their medicines, all children described the size, texture, and taste of their medicines and how these aspects colored their experiences of taking them. For some children there were no issues and the medication was described neutrally.

“they taste of nothingness” (P18)

“They're super small and ok to take” (P16)

However, for many of the children the size, taste, and texture had a negative impact on their experience with the medication.

“they are big and horrible. I can choke” (P17)

“I don't really like it, I don't really like tasting it” (P20)

While many accepted this aspect of the medicine, others found it more problematic. For example, Maisie resorted to hiding or refusing to take her medicine because of how it made her feel when taking it, and afterward.

“Yeah sometimes I hide or I just don’t like taking them because… when you eat you can get that flavor in your mouth and that just.…. sometimes when I have been taking the medicines I haven’t been eating enough food and I have been sick” (P23)

#### 3.5.4 Side effects

Some of the children mentioned the negative side effects they experienced with their medication. For example

“And that one it sorta made me bad because like I just wasn’t acting the same. I went more grumpy and, like, had more fallouts with my Mum” (P3).

“I was taking bad medicine and it made me sick. It was making me really sick and making me have bad emotions and stuff” (P10)

Most children, however, made no mention of side effects of their medication, but were aware of the possibilities. When discussing Ben’s story (comic book vignette), most said that Ben was experiencing the side effect of tiredness

“those [indicates the medicine in the picture] make him tired” (P17)

“it’s his medicine that is doing that […] making him grumpy” (P20).

### 3.6 Children’s understanding of their role in the clinical appointment

In this section, we draw on both the observations of the clinical appointment, and the second interview with children in which the appointment was discussed.

#### 3.6.1 Sharing responsibility with parents

The children discussed their understanding of their role in the appointment. Most children reported that contributions to the appointment should be, shared with their parent(s). Some noted specifically that they had different roles.

“Me and my Mum did it together” (p12)

“Mum answered adult stuff. I did the kid stuff” (P11)

A few children presented themselves as considerably involved in the appointment.

“I did the answering” (P8)

“I think I'd probably ask it anyway because I don't want to, like, leave a question unsaid, you know, like, if I think something's bothering me I should probably say it” (p22)

Asking questions can also be seen as an indicator of children’s involvement in the consultation, however, some children reported that their questions were already addressed by the doctor, meaning that there was no need for them to ask.

“He answered all my questions before I even had to say them!” (P7).

A further way in which some children spoke of being involved was in decision making. For example, when reflecting on a decision to stop his treatment regimen, one boy felt that it was “probably me” who made it. When asked why he made the decision, he replied

“because, like, it's me who has it, I know how I feel and I feel very confident to go off them because I don't feel like I'm going to have one that much” (P3).

A few of the children stated their disinterest in being involved or to contributing in the appointment. For example,

“I just have wee daydreams while I am there” (P13).

Similarly, one girl specified that she did not wish to talk to the doctor about her epilepsy at all, stating that she spoke: “yeah, not much. That’s how I like it”. She later added that she would perhaps think about being more involved, “when I’m older […] kind of like 15, a bit older” (P2).

Some of the children who self-described as minimally involved, suggested that they perhaps ‘should’ be more involved in their appointment. For example,

“I should talk more” (P15).

“I should maybe… I don’t know… it is my appointment” (P4).

Despite this sense of obligation to participate in their appointment, the children expressed some difficulties with being involved.

#### 3.6.2 Not understanding terminology and meanings

As noted above, children’s understandings of epilepsy, and their language for communicating about it, may be different to those of healthcare professionals. There was only one appointment where the consultant specifically asked how the girl and her mother referred to her seizures. The consultant then used the term, ‘moments’, as she described her absences, when asking questions.

Nearly all the children mentioned not understanding “some bits” (P15) of the appointment. For example,

“just really the medication words, they're confusing” (P3),

“[the appointment] makes me feel that I'm not very bright because I don't understand what it means” (P5).

This lack of understanding had implications for some children’s involvement. For example, one girl explained why she chose to stay quiet in her appointment:

P4: because I don’t like to talk

Int: oh no, why not?

P4: I don’t know, I think I’ll just say something wrong

Int: what could you say that would be wrong?

P4: I don’t know I just, something silly

#### 3.6.3 Reluctance to discuss seizures

A further aspect of the appointment that seemed to reduce children’s desire to be involved or to ‘retreat’ from active, verbal participation, were discussions around seizure activity. This relates to some children’s reluctance to discuss seizures with the researcher (3.4.6). During one girl’s (P2) appointment, for example, she initially appeared to be engaged in the appointment, reflecting on her wellbeing and how she was doing at school. However, when the consultant asked about recent seizure activity she looked to her father and then sat very quietly. Her head dropped, and eyes lowered, she avoided looking at anyone, fixing her gaze instead on a mark on the floor and occasionally kicking at it with her feet. Despite her initial contribution the healthcare professional and her father did not ask her further questions or seek to encourage her re-involvement in the discussion. A similar reaction to discussions of seizure activity was observed within many other children’s appointments. One described discussions about her absences as:

“annoying … Just cause… I don’t want to hear about them” (P13).

#### 3.6.4 Feeling silenced by adults

The presence of adults (parents and healthcare professionals) was reported by some children as making it difficult for them to contribute, or left them feeling that their contribution was less valued. In most of the observed appointments, healthcare professionals would frequently ask the child a question – for example, ‘how’s your medication been going?’ – and after hearing the child’s response, would either ask the parent attending the same question or would then look to the parent for confirmation of the child’s answer.

For example from the observation of the appointment, it was clear that one boy had something to say during a particular discussion regarding his school attendance and behavior. At one point, he raised his hand, appearing to try and get the attention of the healthcare professionals or his parents. In response to this, his Mum lowered his hand and leaned in, allowing him to whisper in her ear. No further attempts were made to include him in the conversation, by either the healthcare professional or his parents. When discussing this in the interview that followed, the boy spoke of the challenges of this incident for him to express his views:

P18: I was trying to … I tried to tell him [doctor] that I am getting better behaved than I previously was…

Int: … Do you think he heard you when you were talking?

P18: well I never actually got the word out because I was waiting for when it stopped, for when people had stopped talking for a minute but then of course others were starting again. I didn’t want to interrupt.

### 3.7 Influences on children’s understandings of epilepsy

#### 3.7.1 Information from healthcare professionals

Many of the children recalled receiving written information and resources from a healthcare professional about childhood epilepsy, following diagnosis. For example, one recollected:

“I got a diary and lots of bits of paper telling me about it. Mum got different stuff” (P17).

“I have read all the books about it […] they are just for me” (P18).

As noted in these extracts, the information was designed explicitly for children, and was described by them as “easy to read” (P22). Engagement with these materials appeared to be time-limited, however; most children suggested that they did not look at them beyond the initial diagnosis period.

“Yeah, I think I might still have them, I'm not sure… they were ok to read once” (P22).

#### 3.7.2 Parents as information providers

All the children who participated in this research accounted for their parents, to differing extents, as their primary sources of information regarding epilepsy, for example:

“I talked to my mum about it all the time… she answers my questions about it” (P10).

Several children went on to add how their parents’ knowledge of epilepsy was greater than their own, as one boy stated:

“my mum and dad would know everything about it, not me” (P1).

These extracts illustrate the role of parents as information providers and gatekeepers to knowledge. However, one boy noted that his experiences gave him an insight and expertise that people who do not live with the condition – like his mother – are unable to fully comprehend

“My mum knows a lot, she can explain it to you too. But since I have the fits I would say there might be some hidden things that my mum doesn't know, maybe not know about or something” (P21).

#### 3.7.3 Other children with epilepsy

The children were rarely able to draw on other friends or wider family to enhance their understanding and knowledge of epilepsy; most of the children stated that they had no connection with another child or similar families with epilepsy.

“none of my friends have epilepsy so I can't talk to them about it all” (P22)

Despite this, seeking out others with epilepsy, to meet or talk to about the condition was not mentioned by most children as something they wanted.

## 4 Discussion

This study has explored the way children understand and describe epilepsy. It demonstrated that children tended not to provide medical definitions, or to draw on medical language, but rather spoke about what epilepsy meant by describing the physical sensations of having a seizure or through the act of taking medication. Children described the role they had, or felt they should have, but reported challenges in being meaningfully involved in clinical appointments. While healthcare professionals were initial information nodes, epilepsy information from parents appeared to be more significant for children.

Children’s understanding of epilepsy often draw on their experiential expertise [23], [24]. This included through the use of metaphors about the brain and seizures [25], [26]. Some had difficultly articulating their understanding of epilepsy, in part because they did not *feel* the effect of epilepsy. This may have been because the majority had well-controlled epilepsy (i.e., their seizures had stopped or been greatly reduced as a result of successful treatment regimen). Similarly the children tended to describe their understanding of seizures through physical sensations (or lack of), but also through their emotional response, notably their fear [27], which, for some, led to a discomfort in talking about seizures.

Children’s level of understanding and knowledge of their medication ranged in depth and breadth. While there are gaps in children’s knowledge, their understanding is more than adequate in the context of their everyday life, and should not be seen as a deficiency in awareness or competency, but more as an appreciation of how they have chosen to understand their medication [28]. While there was an awareness and some experience of medication side effects, unlike studies with adolescents, there was less concern expressed about side effects [13]. Instead, the children’s spoke more of their dislike of their medication, leading for some to a reluctance to follow their treatment regimen. Healthcare professionals should seek to understand children’s preferences, identifying any concerns as an important aspect of ensuring medication adherence [29], [30].

Healthcare professionals, who have not done so yet, should acknowledge the legitimacy of children’s experiential expertise and understandings and move away from an adult-centric notion of the ‘expert’ in child health [31]. Despite recognition of the need to involve children in their care, including recognition by the children themselves, children continue to face challenges in being meaningfully and actively involved in healthcare decisions in clinical settings [14], [32], [33]. Parents and healthcare professionals can either enable or impede children’s involvement, depending on whether they appreciate or fail to recognize children’s potential or wish to be involved, and by taking time – or not – to explain aspects of treatment, management, or prognosis were there misalignments in understandings could arise [14], [34]. Healthcare professionals may wish to reflect on the level of communication, and involvement and the style of the interactions being offered and accepted by children and parents within clinic appointments. This is particularly relevant currently given that the COVID-19 pandemic has resulted in a reduction in face-to-face appointments which could result in communication in consultations being limited to patient carers/parents and physicians. Our data highlight the need for the voices of the children themselves to be heard. Facilitating, or strengthening, children’s involvement in appointments requires healthcare professionals to be attentive, sensitive, and supportive of each (individual) child’s expressions, experiences, and perceptions [32].

Changes in clinical practice could be achieved through greater active listening to children’s contributions, engaging them using the words, phrases, and metaphors they use to explain/describe their epilepsy, medication, and seizures. Additionally, greater appreciation of children may change their levels of involvement over time and in relation to different aspects of their condition. Questions, and discussion about seizures, can be particularly challenging for children to be involved in [15]. Awareness of these aspects can ensure that all participants are involved in the appointment as much or as little as they wish.

Clinicians should be increasingly aware of coalitions that can inadvertently form between themselves and parents, potentially isolating the child from being involved or heard. Specifically, although multiple sources of information can provide clinical value the perception of repeating questions to parents must be carefully considered by healthcare professionals given the implications it has for how children feel involved and heard. Where a second perspective from parents is required, it may be useful to consider using different questions, and to ensure that children are still included in the discussion following this line of questioning. Reflecting on these aspects can ensure that children who wish to be actively involved are encouraged to be and are not restricted by communication or engagement barriers.

The current study found that parents were key information gatekeepers: while healthcare professionals were initial nodes in the dissemination of knowledge about epilepsy to children, the information from parents appeared to be a more significant. Moreover, there was minimal opportunity for the children to meet, and so share information with, others with epilepsy. Thus, healthcare professionals should be sensitive to the information needs of children with epilepsy and consider children’s health literacy [35]. A potential solution could be to provide epilepsy-related information in different formats, not just leaflets, which engage children in a manner that is unthreatening, engaging, and provide support to parents in information provision. For example, based on the research, we produced an easily accessible animation about epilepsy that provides information about the condition, seizures, and medication (https://youtu.be/MO7xXL2ZXP8).

## 5 Strengths and limitations

The main strength of this study is that it investigated children’s views directly, through appropriately designed interviews with children. Hightower and colleagues concluded in their study that the insight obtained through interviewing children, could assist healthcare professionals in establishing appropriate and comprehensive help packages to support children with epilepsy [36]. A further strength is the combination of interviews with observation of a consultation. This facilitated an understanding of children’s involvement in discussions during the consultation.

The majority of participants had seizures that were relatively well controlled on monotherapy. The findings in this study cannot therefore be considered to be representative of all diagnoses of epilepsy. Similarly, all participating children had been diagnosed at least two years prior to the interview – again potentially coloring their and their parents’ experiences and reflections on their epilepsy and its care. Children did not have formal cognitive assessment as part of the study, so it is possible that we enrolled children with intellectual disability. However, we consider this very unlikely since experienced, expert physicians screened children and any concerns about possible intellectual disability resulted in study exclusion.

## 6 Conclusions

The perspectives of children with epilepsy are valuable for clinicians to understand; assumptions should not be made that children’s views can be accessed via parents. Clinicians need to be constantly aware of children’s views and ways of understanding and communicating about their epilepsy. To support this, the research – drawing on children’s words, meanings and stories – was used to inform an easily accessible, gender-neutral, animation about epilepsy that provides information about the condition, seizures, and medication.

## Declaration of Competing Interest

The authors declare that they have no known competing financial interests or personal relationships that could have appeared to influence the work reported in this paper.
