# Supplementary material for: Children’s understanding of epilepsy: A qualitative study
Source: Epilepsy Behav. 2021 Jul;120:107994. doi: 10.1016/j.yebeh.2021.107994 (PMC8259124; doi:10.1016/j.yebeh.2021.107994)
Supplement: Supplementary Data 1 [file mmc1.docx]

**Interview Topic Guides and Tools**

**Interview One with Child**

**Experiences/Meanings of Epilepsy**

- Can you tell me what usually happens on an average day?
  - How does epilepsy fit in to a normal day?
  - How does having epilepsy effect a normal day? Or does it?
  - How does having a seizure (or fit, funny turn) change the day? Does it?
- Use Spider Diagram: (a probe for exploring different aspects of the epilepsy experience and the emotions surrounding it).
- What does epilepsy mean?
  - Can you remember when you were first told about epilepsy?
  - What’s a seizure? How do you talk about them?
  - Probe around knowledge of epilepsy
  - Do you take medicines?
    - Do you like taking them?
  - So how do the seizures make you feel?
- Family and Friends?
  - What do your family and friends think about epilepsy?
- What about the future? Will epilepsy affect your future?

**Care Experiences**

- Use family and friends magnets (aim is to uncover who is involved in the epilepsy care, their role within the care, and the feelings associated with this).
  - How do these people help with your epilepsy? What do they do to help you?
- Seizure care
  - Who looks after you if you have a seizure at home?
- What happens when you take your medication?
  - Who helps you take it? Do you do it all by yourself?
- What happens if you don’t take the medication?
- Probe around parental involvement
- Arrange the magnets on the board for friends and school.
  - How do these people help with your epilepsy? What do they do to help you?
- Seizure care?
  - Who looks after you if you have a seizure at school?
  - Do you have medication at school?
  - Who helps you take it? Do you do it all by yourself?

**Comic Book Vignettes**

Three vignettes: a child (Ben) who does not like taking medication, a child (Louise) who decides when to go to bed (child’s responsibility), and a parent (Victoria’s Mum) who does not let her child (Victoria) play outside with friends in case they have a seizure.

- What do you think about that story? What about you? What is it like for you?
- Probe around implications of epilepsy on decision-making (any? None? Lots?)

**Interview Two with Child**

- Imagine you had to tell someone else what happened when you went to the hospital – what would you tell them?
- Use observation notes to probe areas of interest e.g. engagement, participation, and involvement in decisions.
- Pots and beans
  - Who speaks the most? Least?
  - Who makes the decisions? How much say?
  - Who asks questions? Who answers?
  - Discuss how many beans they would want to have in each pot if they could control the appointment.
- Follow up questions from interview one?

**Participatory tools**

- *Spider diagrams:* provide a means to ‘brainstorm’ and discuss thoughts, feelings, and understandings of a particular topic that reach out of the ‘spiders’ legs (Punch, 2007; Johnston, 2008; Johnson et al, 2012). In this study, children were given an A3 sheet of paper with the word ‘epilepsy’ written in a circle in the middle, and each child was asked to draw legs on to this central ‘spider’ with all their thoughts, experiences, and feelings about epilepsy. They could use words, sentences, drawings, or a range of emotion-faces stickers to annotate their diagram, as well as having a wide range of coloured pens. As children added legs to their diagrams, their additions provided a springboard for a more detailed discussion surrounding what they thought of epilepsy, exploring their thinking processes and reflections.
- *Magnetic families and friend’s tool* (Thomas and O’Kane, 1998): was used to explore children’s experiences of, and involvement with the treatment and management of their epilepsy on an everyday basis. Children were given blank magnets, and asked to think of the people in their family who help look after them, and either draw a picture of them or write each person’s name on each magnet, including one for themselves. The magnets were then placed on a board, where they could be moved around. Children were then asked questions about epilepsy and treatment regimens in relation to these family members; for instance, ‘who is involved in their medication?’. The children could then place magnets along the scale of most to least, which then opened up discussion around the ways in which different people were involved.
- *Comic book vignettes*: As stories, vignettes provide concrete examples about individuals, situations, and structures that participants can offer their thoughts on (Braun and Clarke, 2013 Elliott et al 2005). The vignette provides enough context and information for participants to have an understanding of the scenario being depicted, whilst remaining vague in ways to compel participants to ‘fill in’ detail and to answer open ended questions about the story (Jenkins et al, 2010; Braun and Clarke, 2013). The vignettes were presented in the form of a comic strip to be more visually appealing and written in plain English to ensure easy comprehension by children Three vignettes were used to explore experiences of epilepsy and associated treatment and management regimes and decisions that could be typically associated to having and caring for a child with epilepsy. The topics of the vignettes included: Ben, a child, who does not like taking medication; Louise, a child, who decides when to go to bed (child’s responsibility); and Victoria’s Mum, a parent who does not let her child (Victoria) play outside with friends in case they have a seizure. The topics were developed based on observations of clinic appointments and from informal discussions with parents and children with epilepsy to ensure they were realistic. and parents.
- *‘Pots and beads’ ranking exercise:* - this was used in the second interview to explore children’s feelings about the clinic appointment and their involvement in the conversations had, and decisions made. Before introducing the task, children were asked to draw or write on a piece of paper, who was in the room with them. They then chose between different coloured pots to represent each of these individuals and distributed the beads amongst the various pots, from most to least, in response to statements such as: speaking in appointments, asking questions and making decisions. The exercise was used as an interactive tool to engage children in reflexive thought surrounding their own and others’ involvement in clinic appointments rather than to quantify involvement by counting the beads.
